# Describing the Relationship between Cat Bites and Human Depression Using Data from an Electronic Health Record

**DOI:** 10.1371/journal.pone.0070585

**Published:** 2013-08-01

**Authors:** David A. Hanauer, Naren Ramakrishnan, Lisa S. Seyfried

**Affiliations:** 1 Department of Pediatrics, University of Michigan, Ann Arbor, Michigan, United States of America; 2 Department of Computer Science, Virginia Tech, Blacksburg, Virginia, United States of America; 3 Department of Psychiatry, University of Michigan, Ann Arbor, Michigan, United States of America; University of Rennes 1, France

## Abstract

Data mining approaches have been increasingly applied to the electronic health record and have led to the discovery of numerous clinical associations. Recent data mining studies have suggested a potential association between cat bites and human depression. To explore this possible association in more detail we first used administrative diagnosis codes to identify patients with either depression or bites, drawn from a population of 1.3 million patients. We then conducted a manual chart review in the electronic health record of all patients with a code for a bite to accurately determine which were from cats or dogs. Overall there were 750 patients with cat bites, 1,108 with dog bites, and approximately 117,000 patients with depression. Depression was found in 41.3% of patients with cat bites and 28.7% of those with dog bites. Furthermore, 85.5% of those with both cat bites and depression were women, compared to 64.5% of those with dog bites and depression. The probability of a woman being diagnosed with depression at some point in her life if she presented to our health system with a cat bite was 47.0%, compared to 24.2% of men presenting with a similar bite. The high proportion of depression in patients who had cat bites, especially among women, suggests that screening for depression could be appropriate in patients who present to a clinical provider with a cat bite. Additionally, while no causative link is known to explain this association, there is growing evidence to suggest that the relationship between cats and human mental illness, such as depression, warrants further investigation.

## Introduction

The adoption of electronic health records (EHRs) and consequent storage of large quantities of medical data in a computable format has opened the possibility for new discoveries using information that was captured for routine clinical care. Data mining approaches that use powerful data analysis algorithms to unlock hidden patterns in large datasets have been increasingly applied to EHRs. [1] Using such techniques has opened the possibility for uncovering relationships that may ultimately lead to improved health [2], [3].

Data mining the electronic health record has uncovered many potential disease associations,[4]–[9] as well as associations between other aspects of the clinical record including medications and laboratory values. [10] Others have mined EHR data to predict mortality, [11] to detect adverse drug events, [12], [13] and to identify disease-gene associations [14].

In 2009 we reported on an association analysis of clinical concepts that leveraged a tool initially developed for identifying novel gene expressions patterns. [5] That analysis revealed many significant associations among 327,000 patients using free text diagnoses in the problem list of an EHR. While many of the associations were well-known, one intriguing and previously unknown association discovered was between the terms ‘cat bite’ and ‘depression’. The analysis was complicated by the unstructured and unconstrained approach that clinicians used in entering diagnoses.

A follow-up study from 2012 applied a similar analytic approach to a de-identified dataset containing 41.2 million international classification of disease, version 9 (ICD-9) codes from 1.6 million patients. [6] While a specific ICD-9 code exists for dog bites (ICD E906.0), there is no specific code for cat bites. However, one code in particular (ICD E906.3) is most often used to describe cat bites. We again found an association between depression (ICD 311) and animal bites (ICD E906.3), with most of the bites presumably from cats. This additional finding from a much larger dataset suggested that the association between depression and bites was not likely due to chance alone. However, with only a generic code for a variety of animal bites, and no other patient demographic information, little more could be discerned from the data.

Here we report on an extension of the findings from our recent study of ICD-9 codes. [6] Our main objective was to better describe the relationship between cat bites and depression. While the focus of this analysis was on cat bites, we also included dog bites for comparison. We conducted a retrospective chart review of patient records to explore this previously unreported association. We also discuss the potential implications from our findings. By doing so we demonstrate that data mining approaches applied to electronic health records are capable of uncovering novel relationships that can be validated with further study leveraging the data already captured in the EHR.

## Methods

The University of Michigan Health System has had an EHR since 1998, including free text clinical documents created by clinicians through dictation and transcription or by directly typing them into the system. [15] A separate health system data warehouse (HSDW) database contains administrative data such ICD-9 billing codes as well as coded patient demographics. From our institution’s HSDW we identified all patients who were ≥18 years of age with one of twenty-six possible ICD-9 codes for depression (Table 1). We also used the HSDW to identify all patients ≥18 years of age with non-venomous animal bites or injuries. Such bites are represented by the codes E906.X, where X is any of eight categories (Table 2). Venomous animal bites, including bites from certain snakes and lizards, with ICD codes E905.X were not considered in this analysis. We then determined which patients had both depression and a history of a bite. We also characterized the population of patients in terms of age and gender.

**Table 1:** ICD-9 codes used to identify cases of depression.

ICD-9 Code	ICD-9 Code Description	Number of unique patients per code
293.83	Mood disorder in conditions classified elsewhere	1,623
296.2	Major depressive disorder, single episode	370
296.20	Major depressive affective disorder, single episode, unspecified	15,252
296.21	Major depressive affective disorder, single episode, mild	1,811
296.22	Major depressive affective disorder, single episode, moderate	2,957
296.23	Major depressive affective disorder, single episode, severe, without mention of psychotic behavior	2,351
296.24	Major depressive affective disorder, single episode, severe, with psychotic behavior	1,026
296.25	Major depressive affective disorder, single episode, in partial or unspecified remission	1,122
296.26	Major depressive affective disorder, single episode, in full remission	478
296.3	Major depressive disorder, recurrent episode	268
296.30	Major depressive affective disorder, recurrent episode, unspecified	15,557
296.31	Major depressive affective disorder, recurrent episode, mild	3,737
296.32	Major depressive affective disorder, recurrent episode, moderate	9,868
296.33	Major depressive affective disorder, recurrent episode, severe, without mention of psychotic behavior	6,320
296.34	Major depressive affective disorder, recurrent episode, severe, with psychotic behavior	1,670
296.35	Major depressive affective disorder, recurrent episode, in partial or unspecified remission	3,407
296.36	Major depressive affective disorder, recurrent episode, in full remission	1,966
296.9	Other and unspecified episodic mood disorder	8
296.90	Unspecified episodic mood disorder	9,117
296.99	Other specified episodic mood disorder	1,024
298.0	Depressive type psychosis	306
300.4	Dysthymic disorder	10,188
301.12	Chronic depressive personality disorder	17
309.0	Adjustment disorder with depressed mood	9,895
309.1	Prolonged depressive reaction	744
311	Depressive disorder, not elsewhere classified	91,258
Overall*		116,922

* ‘Overall’ represents the unique set of patients for all codes combined, with duplicates across codes removed. Some patients had more than one type of ICD code for their depression and thus were in more than one code category.

**Table 2:** Patients identified with animal bites and injuries based on the E906.X codes, representing non-venomous animal bites and injuries.

ICD-9 Code	ICD-9 Code Description	Unique patientsper code	Non-cat bitepatients	Patients per codewith a cat bite	Additional cat bite patientsidentified (not listed inE906.3)	Total per codewith a dog bite	Additional dog bitepatients identified(not listed in E906.0)
E906.0	Dog bite	1,087	1,061	26	16	1084	N/A
E906.1	Rat bite	27	27	0	0	0	0
E906.2	Bite by non-venomous snakes and lizards	12	10	2	1	0	0
E906.3	Bite by other animal except arthropod (e.g., cats, mice, squirrels, etc.)	866	165	701	N/A	5	2
E906.4	Bite by non-venomous arthropod (e.g., insects)	630	624	6	3	11	6
E906.5	Bite by unspecified animal	174	113	61	8	13	5
E906.8	Other specified injury caused by animal	316	271	45	21	9	8
E906.9	Unspecified injury caused by unspecified animal	56	53	3	2	5	3
Overall*		3,018	2,268	750	49	1,108	24

* ‘Overall’ represents the unique set of patients for all codes combined, with duplicates across codes removed. Some patients had more than one ICD code for their bite(s) and thus were in more than one code category.

N/A = not applicable.

From this cohort of patients with both depression and an animal bite or injury we then conducted a chart review, using a medical record search engine, [16]–[19] of all patients who had any of the E906.X billing codes to determine who had experienced a cat bite. Patients whose documentation described only a cat scratch were not considered to have had a bite. Variations of “bite” such as “bitten” and “bit” were used in the search. Dog bites were found using the same approach.

For patients with a documented cat bite, we recorded the type of relationship between the cat and the bite recipient (i.e, the patient). These were grouped into four categories: (1) Bites from the recipient’s own cat; (2) Bites from the cat of an acquaintance such as a neighbor, friend, or other family member. Bites sustained at a workplace such as a veterinarian’s office were also included in this category; (3) Bites from a stray or feral cat; and (4) Bites where the specific relationship was not mentioned.

Among those patients with either a cat or dog bite we also used the search engine to look through their clinical notes for any social history mentioning if the patient lived alone, suggesting the possibility of social isolation. This was done because nearly 27% of U.S. households (and 28% of Michigan households) are comprised of adults living alone. [20] For this we used the terms “lives” or “living” combined with “alone”, “by himself”, “by herself”, “on his own”, and “on her own”.

All statistical tests were conducted using R for Mac OS X version 2.13.2. Differences in proportions were calculated using the ‘2-sample test for equality of proportions with continuity correction. The University of Michigan Medical School’s institutional review board approved this study, along with a waiver of informed consent. It was determined that the study represented “no more than minimal risk”.

## Results

Out of a total base population of 1.3 million patients, we identified 116,922 patients ≥18 years old with a diagnosis of depression, and 3,018 unique patients who had an animal bite or injury represented by the code E906.X. The most common ICD-9 code for depression was ICD 311, representing 91,258 patients, followed by ICD 296.30 (15,557 patients) and ICD 296.20 (15,252 patients). Tables 1 and 2 report the number of patients per ICD code.

Table 2 also describes a more detailed version of the ICD E906.X patients identified through the chart review. The most common injury code was ICD E906.0 (dog bite) for which there were 1,087 patients, followed by ICD E906.3 (including cat bites and other animals) for which there were 866 patients. Three patients in the dog bite category were misclassified and only had a cat bite, with an additional 23 having had both dog and cat bites. Likewise, there were five patients in the ICD E906.3 category that only had dog bites.

The most common types of bites in the ICD E906.3 category were cats (n = 701, 80.9%), squirrels (n = 45, 5.2%), bats (n = 20, 2.3%) and raccoons (n = 17, 2.0%), although there were a wide variety of animals mentioned in the clinical notes including moles, monkeys, and mice as well as parrots, pigs, piranhas, and prairie dogs. While ICD E906.3 is the traditionally accepted code for cat bites, we identified an additional 49 patients who had experienced a cat bite but were coded in a different ICD E906.X category. Likewise, we identified an additional 27 patients with dog bites that were not coded in the ICD E906.0 category.

Depression rates are shown in Table 3. Among the population of adults with depression, nearly two-thirds were women, although some of this difference may be attributable to more women (54.8%) than men (45.2%) seeking care at the health system. The highest depression rate was for patients who had both a dog bite and a cat bite, with nearly half (47.8%) having depression, all of them women. However, the number of patients with both bites in that category was low (n = 23). Among all patients with a cat bite (n = 750), 41.3% had a diagnosis of depression. This represents a significantly higher rate of depression than the 8.8% rate observed in the general population of adult patients (p<2.2×10^−16^).

**Table 3:** Patients with bites, depression, or both.

		Overall population		Overall bites/injuries (E906.X)		All bites/injuriesexcept cat bites		Cat bites*		Dog bites**		Dog bites (excludingcat bites)		Both dog andcat bites***	
		n (%)	Age, mean± SD	n (%)	Age, mean ± SD	n (%)	Age, mean ± SD	n (%)	Age, mean ± SD	n (%)	Age, mean ± SD	n (%)	Age, mean ± SD	n (%)	Age, mean± SD
Total Patients		1,327,368	54.7±20.5	3,018	42.4±16.6	2,268	40.9±16.1	750	47.0±17.1	1,108	41.8±16.0	1,085	41.7±16.0	23	48.3±15.3
Gender†	F	726,944 (54.8)	54.7±20.7	1,874 (62.1)	43.3±16.7	1,310 (57.8)	41.5±16.3	564 (75.2)	47.3±16.9	572 (51.6)	43.4±16.5	555 (51.2)	43.3±16.5	20 (87.0)	47.6±15.9
	M	600,352 (45.2)	54.6±20.4	1,144 (37.9)	41.0±16.3	958 (42.2)	40.0±15.7	186 (24.8)	45.9±17.7	536 (48.4)	40.1±15.4	530 (48.8)	40.0±15.4	3 (13.0)	53.6±10.9
With depression		116,922 (8.8)	45.5±18.2	957 (31.7)	45.8±16.0	647 (28.5)	45.0±15.6	310 (41.3)	47.5±16.8	318 (28.7)	46.0±16.0	307 (28.3)	45.9±15.9	11 (47.8)	48.4±18.7
Gender	F	76,301 (65.3)	45.4±18.4	714 (74.6)	46.0±16.2	449 (69.4)	45.1±15.8	265 (85.5)	45.1±15.8	205 (64.5)	46.7±16.5	194 (63.2)	46.6±16.4	11 (100.0)	48.4±18.7
	M	40,620 (34.7)	45.8±17.9	243 (25.4)	45.2±17.6	198 (30.6)	44.7±15.0	45 (14.5)	44.7±15.0	113 (35.5)	44.8±15.0	113 (36.8)	44.8±15.0	0 (0.0)	–

* Includes patients with an identified cat bite from all categories, not just E906.3.

** Includes patients with an identified dog bite from all categories, not just E906.0, as well as 23 patients who had both a dog and a cat bite.

*** Includes only patients who had both a dog and a cat bite, although not necessarily on the same day.

† 72 patients had an “unknown” gender (<0.01% of overall population).

The 41.3% depression rate among those with a cat bite is also higher than those who received any kind of bite or injury other than a cat (28.5%, p = 8.7×10^−11^), including dog bites (28.7%, p = 2.2×10^−8^). In fact, the proportion of men and women with depression having dog bites closely mirrored the gender proportion of depression in the general population, whereas this was not the case with cat bites. Among the 310 patients who had a cat bite and depression, 85.5% were women. This compares to 64.5% women among those who had a dog bite and depression, which is similar to the overall percentage of women in the depression population (65.3%). To state this differently, based on over 10 years of data, if a woman presented to our health system with a cat bite that was serious enough to be coded as such, there was a 47.0% chance that she will also be given a diagnosis of depression at some point in her life. For men, about half as many (24.2%) had depression. By contrast, the gender difference was still present but smaller for dog bites: if a woman presented with a dog bite, there was a 35.8% chance of having depression compared to 21.1% of men.

Regarding living situation, 204 out of 750 (27.2%) patients with cat bites were living alone compared 178 out of 1,108 (16.1%) patients with dog bites (p = 8.0×10−9). However, among those living alone there was no significant difference in depression rates, with 125/204 (61.3%) of cat bite patients and 104/178 (58.4%) of dog bite patients living alone having a diagnosis of depression (p = 0.64).

**Figure 1:**
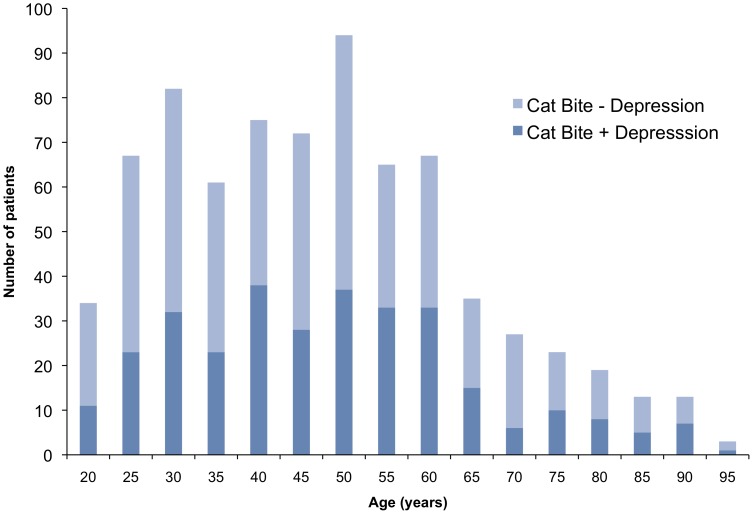
Stacked bar chart showing age versus total number of patients, for 750 patients with cat bites. Dark blue bars represent patients with depression and light blue bars represent patients without depression. Ages are rounded to the nearest 5-year period.

Age was not a major factor in our results, with very little difference in ages among those with or without depression, or by gender. Figure 1 shows a histogram displaying the ages of the patients who experienced a cat bite, divided into those with and without depression. More than half (58.5%) of the patients who had a cat bite were under the age of 50. The most common decade of life for patients to have experienced a cat bite were for patients age 40–49 (n = 163), followed by 50–59 (n = 156), 30–39 (n = 139), and 20–29 (n = 128). Notably, there were far fewer patients ages 60–69 (n = 76) with a cat bite, and even fewer bites among the older patients.

Because some patients may have presented to the health system initially with a bite and only later were diagnosed with depression, we also looked at the temporal occurrence of bites with respect to depression diagnoses, shown in Table 4. There was a slightly significant difference in the proportion of patients being diagnosed with depression first. Whereas about a quarter (27.1%) of patients with cat bites and depression first had a cat bite followed by depression, about a third (36.2%) of dog bite patients had a bite that preceded the depression (p = 0.02).

**Table 4:** Temporal relationships among the time of first diagnosis of depression and cat or dog bites.

		Patients with Cat Bites and Depression (n = 310)			Patients with Dog Bites and Depression (n = 318)		
		Depressiondiagnosed first	Bite diagnosed first	Diagnosed on same day	Depressiondiagnosed first	Bite diagnosed first	Diagnosed on same day
		n (%)	n (%)	n (%)	n (%)	n (%)	n (%)
Total patients		220 (71.0)	84 (27.1)	6 (1.9)	196 (61.6)	115 (36.2)	7 (2.2)
Gender	F	190 (86.4)	70 (83.3)	5 (83.3)	138 (70.4)	63 (54.8)	5 (71.4)
	M	30 (13.6)	14 (16.7)	1 (16.7)	58 (29.6)	52 (45.2)	2 (28.6)

The majority (58.8%) of bites in our study were inflicted by the patients’ own cat, including 56.1% among those who had depression (Table 5). Being bitten by a stray or feral cat was least common (15.7% of all bites, and 15.2% of bites among those with depression). However, among those with depression who had bites from stray or feral cats, women far outranked men (93.6% vs 6.4%, respectively).

**Table 5:** Background of the biting cat, categorized by gender and depression of the human bite recipient.

		Own Cat	Acquaintance Cat	Stray Cat	Not Reported
		n (%)	n (%)	n (%)	n (%)
Total Patients (n = 750)		441	146	118	45
Gender	F	329 (74.6)	112 (76.7)	93 (78.8)	30 (66.7)
	M	112 (25.4)	34 (23.3)	25 (21.2)	15 (33.3)
With depression (n = 310)		174 (39.5)	69 (47.3)	47 (39.8)	20 (44.4)
Gender	F	141 (81.0)	58 (84.1)	44 (93.6)	15 (75.0)
	M	33 (19.0)	11 (15.9)	3 (6.4)	5 (25.0)

## Discussion

Depression remains a significant public health concern, has been linked to increased mortality, and is predicted to be one of the three leading causes of illness worldwide by 2030. [21] In the United States depression remains one of the top causes of disability, especially among females. [22] The national prevalence of 12-month and lifetime major depression, has been estimated to be 8.1% and 18.6%, respectively. [23] Yet, depression treatment can yield reasonable response rates if adequate follow-up occurs [24]–[26].

Screening interventions to detect depression, although somewhat controversial, [27]–[30] are often advised. [28], [31], [32] A small study from 1995 reported that primary care physicians may miss major depression in their patients up to 40% of the time. [33] Current detection rates might be higher in settings such as the Veterans Health Administration where yearly depression screening is now a requirement, [34] although routine depression screening is not universally done elsewhere [35].

While universal depression screening might be ideal, such screening will likely not occur in all medical settings, and targeted screening may be preferable with limited time and resources. [36] It may seem counterintuitive to consider screening for depression in someone who presents to a clinician with an acute injury from a household pet, but our findings suggest that it could be beneficial. Table 6 represents a reformulated subset of data from Tables 3 and 5 and reports on the probability of having depression given a patient’s gender and type of bite. These data demonstrate that depression seems to be higher in all patients with bites compared to the general population. Additionally, it suggest that cat bites, especially in women, might serve as a warning sign for depression. Depression has been identified in other populations using various criteria for detection. For example, it has been suggested that adolescents presenting to the emergency department with non-specific somatic complaints such as chest pain or headaches could have depression, [37] which should prompt further screening. [38] Similarly, patients presenting to the emergency department or cardiology clinic with chest pain may actually have a panic disorder. [39], [40] In our study population, the quarter of patients with cat bites that preceded their depression and the third of patients with dog bites that preceded their depression potentially represent a population of patients for which screening at the time of the bite might have detected the depression sooner. The literature is already replete with reviews that alert physicians to consider infections when patients present with cat bites, [41]–[43] so it may be reasonable that depression also be considered.

**Table 6:** Percent probability of having depression given the following conditions.

		All patients	Dog Bite	Cat Bite (all cats)	Cat Bite (own cat)	Cat Bite (acquaintancecat)	Cat Bite (strayor feral cat)
Gender	F	10.5	35.8	47.0	42.9	51.8	47.3
	M	6.8	21.1	24.2	29.5	32.4	12.0

For example, the probability of depression given that a woman presents with a bite from her own cat is 42.9%.

Our study is not the first to have reported on the high prevalence of cat bites in women.[44]–[48] Several studies have reported an approximately 2-to-1 ratio of women to men with respect to cat bites, [49]–[52] a proportion not seen with dog bites. In our study population the ratio of women to men with cat bites was approximately 3-to-1. Another study reported that bites (including cat bites, but excluding dog bites) were ranked sixth in terms of frequent causes of unintentional injuries in adult women. [53] An association between pet ownership (dogs and cats) and depression has previously been reported among women but not men [54].

The finding of a strong association between cat bites and depression, while intriguing, still does not point to why such a connection exists, and the relationship is likely complex. Here we discuss potential factors that may play a role, but emphasize that our study was not designed to elucidate the underlying mechanisms for why the association exists. Furthermore, such an association does not necessarily imply causation.

There is substantial evidence to suggest that pet ownership results in multiple health benefits, both physical and mental [55]–[69]. For example, pet ownership has been shown to reduce elevated blood pressure caused by mental stress even better than antihypertensive medications. [70] Pets can also provide substantial social support. [63] A study in Switzerland reported that among people living alone, cats could improve their mood. [71] As such, it may be that depressed individuals, especially women, are more likely to own cats for companionship. [72] Pet ownership has also been shown to moderate the effect of depression on mortality in patients who experienced a myocardial infarction [60].

But not all studies have reached similar conclusions, and the role of pets and human health remains controversial with multiple studies reporting inconclusive results [54], [59], [65], [66], [73]–[80]. For example, one study found a substantial survival benefit after myocardial infarction for dog owners but not cat owners. [74] It is also important to note that many health studies that considered pet ownership did not distinguish between cat and dog ownership.

A study in Canada reported that those living alone with a cat were just as lonely and depressed as those living alone without a cat, suggesting that the cat itself was not contributing significantly to an improved sense of well-being. [81] In this group, having high levels of social support decreased loneliness for dog owners but not for cat owners. Additionally, among those with low levels of social support, those who were more attached to their pets (both dogs and cats) had more depression than those with less attachment, a finding which has been corroborated elsewhere. [82] Another study reported that adults in Australia ages 60–64 with pets (dogs or cats) had more depression symptoms than non-pet owners [83] and a large study of Finnish adults found a small but significant increase in depression among those who owned a pet (12.6%) versus no pet (11.3%). [54] In one case the stress of caring for pet cats was a contributing factor in worsening a patient’s depression. [68] Other studies have shown no benefit to pet ownership among women in terms of reducing loneliness [72] or reducing emotional distress [84].

Perhaps most intriguing is the possibility that *Toxoplasma gondii*, a parasite commonly found in cats and known to infect humans, could be causing long-term changes to the cat owners’ brains. Recent studies have suggested that this parasite may actually contribute to human psychiatric disorders such as schizophrenia and obsessive compulsive disorder, as well as other brain pathologies [85], [86] and personality changes [87]–[90]. The *Toxoplasma* parasite has been linked to prenatal depression, [91] and a case report from 2004 discussed a patient with depression who was not responsive to anti-depressant medications until the parasite was eradicated. [92] Infections from the parasite have been associated with self-inflicted violence [93], [94] as well as increased suicide rates in women. [95] It has also been suggested that the inflammatory cytokines released during a *T. gondii* infection in the brain may be the cause of depression in some patients [96], [97].

The prevalence of *T. gondii* seroposivity in the United States has been dropping, but has been estimated to be around 11%. [98] It should be noted that cat bites, per se, are not thought to transmit the parasite. Rather, the parasite is typically shed in the feces of cats. Most transmission occurs orally through contact with contaminated food or drink, or in utero from mother to fetus. This is why pregnant women are advised not to change a cat’s litter box [99].

Not all evidence points to *T. gondii*, or their cat hosts, as being relevant to the depression relationship, however. The seroprevalence of *T. gondii* is generally higher in men than in women, [98], [100] and one study found that cat ownership was not a significant risk factor for being seropositive for the parasite. [100] Because of the nature of how the parasite is transmitted, it has been suggested that exposure to cats may not even be a significant risk for infection. [101] Indeed, some studies have not found a strong link between cat exposure and having an infection from or antibodies to *T. gondii*, [102], [103] with one study reporting a correlation only for those who had 3 or more kittens, but not for those with fewer. [104] Furthermore, while cats are necessary for the life cycle of the T. gondii parasaite, dogs have also been implicated in its transmission to humans [105].

Another interesting aspect of our findings is how they were discovered–that is, a non-directed, non-hypothesis driven data mining algorithm uncovered this unusual association between cat bites and depression. Further, we were able to corroborate the initial findings with a retrospective chart review. All of this was done using data that had been collected over the course of many years, by many clinicians, none of whom would likely have detected this pattern or relationship on their own. This secondary use of pre-existing clinical data is one component of the proposed ‘Learning Health System’, in which electronic data from institutions around the country, and potentially the world, could be pooled with the goal of enhancing discovery and improving patient outcomes. [106], [107] Others have also made discoveries through data mining approaches that were further confirmed with analyses of EHR data [108], [109].

There are challenges of finding these potentially novel associations among the many hundreds of thousands that are uncovered with large-scale data mining approaches, in part because the most significant associations are already known. [6] It is therefore necessary to search through the many less significant associations to detect new findings. In unpublished data from our original study of free text associations, the term ‘depression’ ranked 20 out of the 117 significant associations with ‘cat bite’, whereas ‘cat bite’ ranked 1,898 out of 3,879 significant associations with depression. [5] In the follow-up study of ICD-9 codes, ICD 311 (depression) ranked 26 out of 723 associations with ICD E906.3 (animal bite), whereas ICD E906.3 ranked 1,296 out of 6,667 among association with ICD 311 [6].

Our study does have several limitations, some of which are likely inherent in any large-scale analysis of EHR data. [110], [111] Like any retrospective study, we were limited by what was (or was not) reported by the clinicians in their clinical notes, or by which patients chose (or did not choose) to seek medical treatment. One study reported that nearly 40% of people with cat bites did not seek medical care. [46] It is also possible that our results could be skewed if depressed patients are more likely to seek care for a bite; depressed patients without a supportive social network may be more likely to be seen by a doctor for issues such as bites. While we attempted to determine who lived alone, we did not assess how much social support each individual had.

Additionally, there are many confounding factors other than the higher prevalence of depression in women that might influence the context in which our findings should be interpreted, Such factors might include (1) the age of the cat; (2) the number of cats or pets in the household; (3) the living arrangements of the cat (e.g., indoor vs outdoor) (4) the length of time the cat and bite recipient knew each other; (5) the events occurring at the time of the bite (e.g., taking the cat to the veterinarian, breaking up a fight between two cats, giving the cat a bath, accidentally stepping on the cat); (6) the cat’s temperament and prior history of biting; (7) the health status of the cat; and even (8) the profession of the patient. Some of the bites were noted to happen with people working in animal shelters, pet stores, or veterinarian offices. Women are now entering veterinary medicine professions more than men, [112], [113] and women more than men veterinarians often work in small animal practices (i.e., those that treat cats as opposed to cows) [114].

The preponderance of cat bites in females may also be due to a preponderance of women owning, or caring for, cats. One study from the American Pet Association reported that 80% of cat owners were women [115] whereas another from Gallup survey from 2007 reported no gender differences in cat or dog ownership. [116] According to 2006 data from the American Veterinary Medical Society (AVMS), women are more often the primary caretakers of cats as compared to men (78.1% vs 21.9%). [117] What is harder to explain is that in our study nearly an equal proportion of women and men suffered from dog bites, yet according to the AVMS women are also more often the primary caretaker of dogs as compared to men (74.2% vs 25.8%). Furthermore, among those living alone, pet ownership of cats and dogs is roughly equal: 13.1% of single-occupant households own a dog only, 15.9% own a cat only, and 5.4% own a cat and dog. How these single-occupant households with pets differed by gender is unknown, but according to recent U.S. census data there are slightly more single households with women (14.6%) than men (11.2%) [20].

Compared to men, women have been reported to own cats more often in other countries as well including the United Kingdom (UK). [118], [119] One study from the UK reported that 27% of women and 20% of men owned a cat. [120] Another study from the UK reported women to be 3.5 times more likely than men to own a cat. [121] Women are about twice as likely than men to own dogs in the UK, [118], [119] although other studies have reported a smaller difference in dog ownership (24% of women and 22% of men, respectively) [120].

It has also been suggested that there are personality types associated with cat or dog owners. [122] One study reported that women comprised 68.1% of those who defined themselves among the “cat people” group and 58.6% among the “dog people” group. [123] Those in the “cat people” group tended to have higher levels of neuroticism compared to “dog people” based on personality testing. Interestingly, though, there were similar levels of neuroticism among men and women who defined themselves as “cat people”, whereas a larger difference existed between the genders among “dog people”. The neuroticism personality trait has been strongly associated with depression, [124], [125] although whether neuroticism represents a vulnerability for developing depression is unclear [126].

Some animals may bite more in response to changes in their owners' mental state or level of responsiveness. For example, depressed individuals often make less eye contact compared to those without depression. [127], [128] Some animals, such as dogs, horses, and pigs are known to respond to human social cues such as gestures, gaze, and focus.[129]–[133] Even cats may respond to respond to pointing gestures [134] and human gaze. [135] One study reported that the type of activity being undertaken by a test subject had a large impact on a cat's behavior. [136] And another study of individuals living alone with a cat found differences in interactions with cats that were correlated with self-reported moods of “depressiveness” [137].

It is also possible that the risk of bites is greater in homes with multiple pets. Breaking up fights between cats was noted in the medical record to be a reason some patients were bitten by cats. Households are more likely to own multiple cats as opposed to multiple dogs. A recent AVMS survey reported that the mean number of dogs per household was 1.7, and 37.8% of dog owning households had more than one dog. Of cat owning households the mean number of cats was 2.2, and 51.8% had more than one cat. Additionally, 41% of dog-owning households had at least 1 cat and 47% of cat-owning households had at least one dog. [117] Another study reported that 13% of UK households owned 1 cat and 11% owned 2 or more cats whereas 17% of UK households owned one dog and 5% owned more than one dog [120].

Generalizability may also be an issue since the patients and pets that are predominant in Southeastern Michigan may not reflect the makeup of patients or pets in other areas of the country, including more rural or urban settings. For example, a study of over 6,000 bites occurring in New York City reported about 70% dog bites and 13% cat bites. [138] In our dataset of bites using the ICD E906.X codes, dogs made up 37% of bites and cats about 25%. We also do not know what the overall prevalence of pet ownership was among our patient cohort. Household pet information is rarely captured in the EHR except for when it might impact on allergic symptoms. However, the overall ownership level by household of dogs (36.8%) and cats (32.5%) in Michigan closely matches the national average (37.2% dogs, 32.4% cats) [117].

Another limitation of our study is that ICD-9 codes are used primarily for billing purposes rather than clinical care, and these codes are often inaccurate, [139]–[141] including for depression. [142] This was evident even in our study in which dog and cat bites were misclassified among multiple ICD-9 categories. We attempted to compensate for inaccurate coding by conducting a chart review of the patients’ records with bites. We did not, however, confirm the depression diagnosis among the patients who had one of the ICD-9 codes representing depression. Further, we focused our search on patients who had an ICD-9 code for an animal bite, but there may have been other instances in which the bite was coded differently (e.g., as an open, penetrating, or puncture wound instead). The United States will shortly migrate from ICD-9 to ICD-10 codes, the latter of which does have a set of codes specific to cat bites (e.g., W55.01), which may make such analyses more accurate.

Nevertheless, our study did include a base population of over one million patients with over ten years of data collected in the EHR. While the total number of patients with cat bites in our study was relatively small, the consequences of untreated depression can be large. It may be that the relationship between cat bites and human depression is spurious and no true cause-and-effect exists. But if the relationship can be shown to hold true in other settings it suggests that, whatever the underlying reason, depression should be considered by health care practitioners when patients, especially women, present with cat bites. For at least a subset of these patients, the presentation of cat bite may be their initial contact with a healthcare provider, and screening these patients for depression could provide a means for early detection. Further research is needed to better understand this unusual relationship. We believe that, at the least, this study demonstrates that leveraging the power of data mining with follow-up chart reviews has the potential to improve health. As clinicians continue to collect electronic data that can be aggregated and explored with various data mining approaches, the possibilities for research and data discovery will continue to grow.
